# GoFlow: efficient transition state geometry prediction with flow matching and E(3)-equivariant neural networks

**DOI:** 10.1039/d5dd00283d

**Published:** 2025-10-21

**Authors:** Leonard Galustian, Konstantin Mark, Johannes Karwounopoulos, Maximilian P.-P. Kovar, Esther Heid

**Affiliations:** a Institute of Materials Chemistry, TU Wien A-1060 Vienna Austria esther.heid@tuwien.ac.at

## Abstract

Transition state (TS) geometries of chemical reactions are key to understanding reaction mechanisms and estimating kinetic properties. Inferring these directly from 2D reaction graphs offers chemists a powerful tool for rapid and accessible reaction analysis. Quantum chemical methods for computing TSs are computationally intensive and often infeasible for larger molecular systems. Recently, deep learning-based diffusion models have shown promise in generating TSs from 2D reaction graphs for single-step reactions. However, framing TS generation as a diffusion process, by design, requires a prohibitively large number of sampling steps during inference. Here we show that modeling TS generation as an optimal transport flow problem, solved *via* E(3)-equivariant flow matching with geometric tensor networks, achieves over a hundredfold speedup in inference while improving geometric accuracy compared to the state-of-the-art. This breakthrough increase in sampling efficiency and predictive accuracy enables the practical use of deep learning-based TS generators in high-throughput settings for larger and more complex chemical systems. Our method, GoFlow, thus represents a significant methodological advancement in machine learning-based TS generation, bringing it closer to widespread use in computational chemistry workflows.

## Abstract 2

We introduce GoFlow, a package to generate 3D transition state geometries using flow matching.

## Graphical abstract

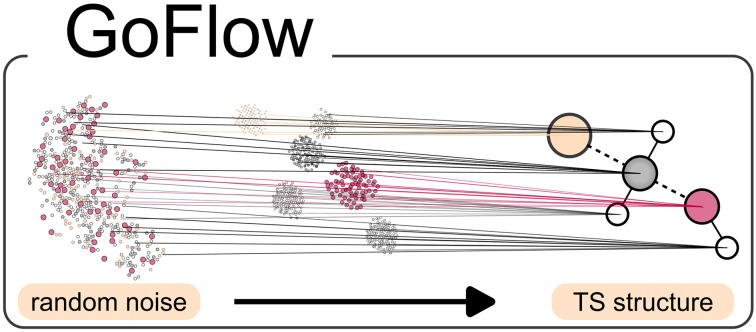

## 1 Introduction

Transition states (TS) of chemical reactions determine the activation energy and consequently the reaction rate. They are saddle points on the potential energy surface of a reaction and lie on the minimum energy path (MEP) between reactants and products.^1,2^ Knowing their geometry is crucial for understanding reaction mechanisms and identifying dominant pathways in reaction networks. Thus, providing chemists with an efficient, accurate, and user-friendly method capable of determining the TS structure directly from the 2D reaction graph would significantly accelerate laboratory research and workflows, and thereby reduce costs.

TSs are high-energy structures that are extremely short-lived and exist only on the order of femtoseconds.^3^ To better understand chemical reactions, for decades, quantum mechanical calculations have been the only way of accessing TS structures. Density functional theory (DFT) has commonly been used as the method of choice.^4^ TS search algorithms generally fall into two categories: single-ended methods mostly start from the 3D geometries of the reactants,^5^ while double-ended methods utilize both reactant and product geometries for determining the TS.^6^ These quantum chemical methods are, however, computationally highly demanding, for example, using ≈35% of the computational resources of the Swiss National Supercomputing Center in 2017.^7^ They also suffer from convergence difficulties, often yielding TSs that do not lie on the minimum energy pathway.^8^

Several reaction datasets containing optimized TS geometries have been developed in recent years.^8–13^ One notable example is RDB7,^9^ which builds on the work of Grambow *et al.*^10^ In their approach, small organic molecules were sampled as reactants from GDB-7,^14^ and their optimized geometries were used as starting points for a growing string method and subsequent saddle point optimization to identify TSs. The main aim of such reaction datasets is to enable researchers to develop machine learning (ML) based algorithms for estimating TS geometries and barrier heights. ML algorithms identify patterns from thousands of chemical reactions that are predictive of the TS geometries, and thus avoid running expensive quantum chemical calculations.

Multiple deep learning (DL) based algorithms have been proposed for predicting TS structures. Some of them rely on the 3D geometry of the reactant and product as input, in addition to the 2D graphs,^15,16^ while others take only the 2D graph as input.^17,18^ Generating 3D geometries of reactants and products can be computationally costly, with current studies relying on external quantum chemistry calculations that provide relaxed 3D coordinates.^15,16,19^ Furthermore, current methods perform post-processing using quantum chemical methods on the generated TS structures.^15,17,18^ In both cases, high-throughput applications cannot rely on these expensive calculations.

Kim *et al.*^18^ recently introduced TsDiff, the current state-of-the-art (SOTA) approach for generative modeling of TS geometries leveraging only the 2D reaction graph as input, encoded as a condensed graph of reaction.^20^ TsDiff relies on diffusion modeling^21^ of the distribution of possible TS geometries with an E(3)-invariant, distance-based graph neural network (GNN) as encoder.^18^ While achieving remarkable results, framing the problem as a diffusion process requires a large number of denoising steps during inference. TsDiff in particular uses annealed Langevin dynamics for inference, requiring 5000 sampling steps per reaction, considerably slowing down inference. Duan *et al.*^16^ have recently shown improved TS prediction performance on the Transition1X (T1X) dataset^12^ using a deterministic optimal transport based model that, given a TS structure guess, produces a single TS prediction. However, deterministic methods require knowledge of the reactant and product geometries, which correspond to a particular TS, which we do not assume.

Another important drawback of current deep learning approaches for the generation of TS coordinates arises from overfitting and lack of generalization capabilities. Previous studies found GNNs and, in general, DL-based reaction prediction models to consistently generalize poorly,^8,22^ or perform significantly worse on several possible out-of-distribution dataset splits,^8,19^ even though the test reactions shared many common reaction mechanisms with the training reactions.^8,23^

In this paper, we address the current limitations of efficiency, necessity of additional quantum-mechanical calculations, and missing out-of-distribution generalization ability. Specifically, we frame the TS structure generation problem as an optimal transport flow process with an E(3)-equivariant geometric tensor network^24^ as graph encoder, taking as input only the 2D reaction graphs. Using a flow matching-based algorithm^25^ for fitting the velocity field, and the efficiency of geometric tensor networks, increases sampling efficiency during inference by more than a hundredfold, compared to the SOTA. Second, we analyze out-of-distribution generalization capabilities of our generative model using splits based on the clustered reaction cores and the magnitude of the barrier height, in addition to random splitting. Current literature for TS prediction using DL has so far only used random splits.^15–18^ We demonstrate that our approach, GoFlow, outperforms previous methods in terms of generalization capability. Together with its improved in-distribution performance at a fraction of the cost of current approaches, we establish GoFlow as the new state-of-the-art in generative TS models.

## 2 Methods

### 2.1 Problem setup

Given the 2D graphs of reactant and product molecules of single-step reactions, our objective is to predict the most likely TS geometry of the reaction. The 2D reaction graphs are given in SMILES notation.

Initial atom and bond features ({**h**_*i*_}_*i*_,{**e**_*j*_}_*j*_) are extracted using RDKit.^26^ The model outputs cartesian coordinates 
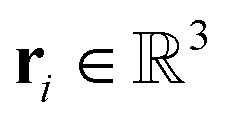
 per atom *i*.

### 2.2 Condensed graph of reaction

We encode chemical reactions using the condensed graph of reaction (CGR).^20,27,28^ The CGR is a representation that encodes chemical reactions as a single graph by superimposing the molecular graphs of reactants and products. Each atom and bond in the CGR carries dual labels, indicating its state before and after the reaction, which allows the model to capture changes in bonding, charge, hybridization, *etc.*

It has been shown to be highly effective for both TS geometry prediction,^18^ and reaction property prediction.^20,29^

### 2.3 Flow matching

Let *p*_0_(*x*) be an easy-to-sample distribution, in our case 
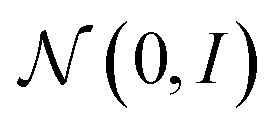
, and *p*_1_(*x*) be the target data distribution that we want to model. Flow matching considers a continuous path of probability distributions *p*_*t*_(*x*) for *t* ∈ [0, 1] such that *p*_*t*_ smoothly interpolates between *p*_0_ and *p*_1_. This path is induced by a time-dependent vector field *v*_*t*_(*x*). Samples *x*_*t*_ evolving along this path follow the ordinary differential equation (ODE):^30^
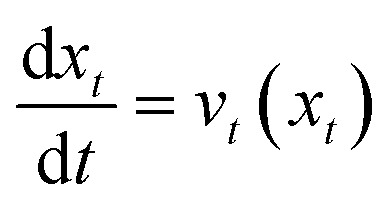
where *x*_*t*_ ∼ *p*_*t*_. The vector field *v*_*t*_(*x*) and the probability path *p*_*t*_(*x*) are linked *via* the continuity equation:∂_*t*_*p*_*t*_(*x*) = −∇ × (*p*_*t*_(*x*)*v*_*t*_(*x*)).

The goal is to train a parameterized neural network *u*_*θ*_(*x*, *t*) to approximate the true, often intractable, vector field *v*_*t*_(*x*).

A key challenge is that *v*_*t*_(*x*) depends on the marginal probability path *p*_*t*_(*x*), which is typically unknown. Instead of directly matching the marginal vector field *v*_*t*_(*x*), conditional flow matching (CFM) defines a simpler target vector field based on conditional paths.^30^

Consider a specific pair of samples *x*_0_ ∼ *p*_0_ and *x*_1_ ∼ *p*_1_. We can define a path *x*_*t*_ connecting them, for instance, a simple linear interpolation, also called the optimal transport (OT) path:*x*_*t*_ = (1 − *t*)*x*_0_ + *tx*_1_.

The vector field corresponding to this specific conditional path is simply the time derivative
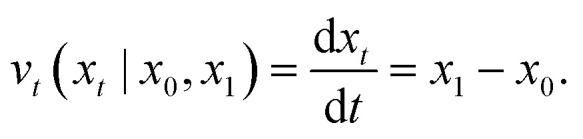


Crucially, this target vector field (*x*_1_ − *x*_0_) is independent of *t* and *x*_*t*_ along the path and does not require knowledge of *p*_*t*_(*x*).

The OT CFM objective trains the model *u*_*θ*_(*x*, *t*) by minimizing the expected squared error against this conditional target vector field, averaged over time *t* and pairs (*x*_0_, *x*_1_):
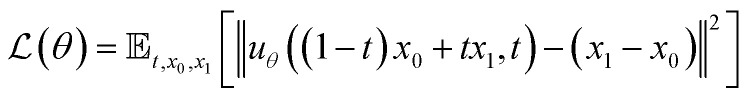
with 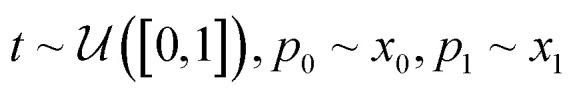. Minimizing this objective results in *u*_*θ*_(*x*, *t*) approximating the marginal vector field *v*_*t*_(*x*).^25^

Once the model *u*_*θ*_(*x*, *t*) is trained to approximate the vector field *v*_*t*_(*x*), it can be used for generating new samples from the target distribution *p*_1_(*x*). This is achieved by numerically simulating the probability flow ODE. Starting with an initial sample *x*_0_ drawn from the base distribution *p*_0_(*x*), we integrate the differential equation
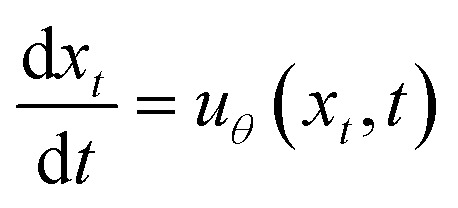
forward in time from *t* = 0 to *t* = 1. Standard numerical ODE solvers, such as Euler or Runge–Kutta methods, can be employed for this simulation; in this work, we employ Euler's method. The resulting state *x*_1_ at *t* = 1 is then considered a sample approximating the target distribution *p*_1_(*x*).

### 2.4 E(3)-equivariance

Capturing molecular 3D structure and symmetry is crucial for data-efficient prediction of properties like energy, forces, and dipoles.^31,32^ E(3)-equivariant GNNs achieve this by respecting the symmetries of the Euclidean group E(3), namely 3D rotations, reflections, and translations.^33^

Let *X* = ({**r**_*i*_}_*i*_,{**h**_*i*_}_*i*_) represent the input molecule, with coordinates 
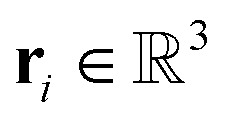
 and initial features **h**_*i*_. Let *Φ* be the network function mapping *X* to output features **F** = *Φ*(*X*). An E(3) transformation *g* ∈ E(3) acts on the coordinates as *g* × **r**_*i*_. We denote the transformed input as *g* × *X*=({*g* × **r**_*i*_}_*i*_,{**h**_*i*_}_*i*_).

Let *V* be the space of output features **F** and GL(3) the group of invertible linear transformations on it. The network *Φ* is E(3)-equivariant if there exists a representation *ρ*: E(3) → GL(*V*) such that for all *g* ∈ E(3) and all inputs *X*:*Φ*(*g* × *X*) = *ρ*(*g*)*Φ*(*X*).

This means that transforming the input geometry by *g* results in a predictable transformation *ρ*(*g*) of the output features. For instance, rotating the input molecule causes the predicted force vectors to rotate accordingly.

Scalar outputs, such as energy, must be E(3)-invariant, a special case where *ρ*(*g*) is the identity transformation, *ρ*(*g*) = **I**, for all *g*.

Implementations often use spherical harmonics (*e.g.* up to *L* = 2) as bases for features, which transform *via* Wigner D-matrices under O(3) rotations. Tensor products are key operations for maintaining equivariance. Examples include SE(3)-Transformers, NequIP, and MACE,^31,34–36^ known for their expressiveness but potentially high computational cost.

In this work, we adapt and use the E(3)-equivariant Geometric Tensor Network (GotenNet) architecture.^24^ GotenNet aims to bridge this gap between expressiveness and efficiency, particularly addressing the computational overhead associated with traditional tensor product based methods. It achieves E(3)-equivariance without explicitly relying on tensor products with Clebsch–Gordan coefficients for its core message passing.

GotenNet works with different types of features, capturing geometric information. Nodes have invariant scalar features *h*_*i*_ and steerable features *X̃*^(*l*)^_*i*_ that behave as spherical harmonics up to a degree *L*_max_. Edges also have invariant scalar features *t*_*ij*_ and initial geometric tensors *r̃*_*ij*_ derived directly from the relative positions of connected atoms using spherical harmonics.

Specifically, the authors introduce multiple equivariant modules, such as geometry-aware tensor attention and hierarchical tensor refinement. They modify transformer-based architectures by refining edge representations through high-degree steerable features, which enable the attention mechanism to leverage geometric relationships in determining node interactions. For details, we refer the reader to the paper.^24^ The key point is that GotenNet does not use high-degree tensor product operations, thus improving efficiency, while still capturing essential geometric information.

### 2.5 Sample aggregation

We introduce a novel aggregation method that reduces variance and significantly improves the accuracy of predicted geometries, without requiring changes to the training algorithm.

During inference, we sample multiple TS geometries for each reaction. Let *S* be the number of samples and let 
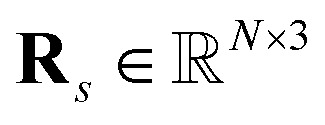
 denote the matrix of atomic coordinates for the final geometry of the *s*-th sample, where *N* is the number of atoms. The coordinates for the *i*-th atom in sample *s* are 
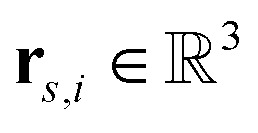
.

To choose the final prediction, we first compute the median atomic position **r̄**_*i*_ for each atom *i* across the *S* samples, where atoms are identified by their atom mapping number:
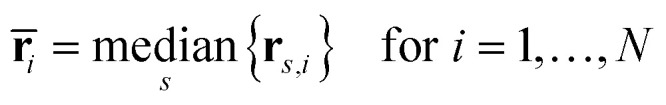


These median positions form the aggregate median geometry 
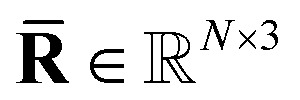
.

Finally, we choose the sample 
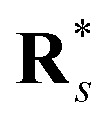
 whose geometry is closest to the median geometry **R̄**. The distance *d*_*s*_ for each sample is calculated as the Frobenius norm of the difference between the sample's coordinates and the median coordinates:
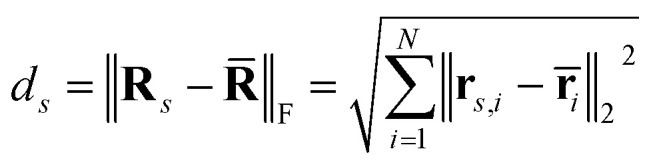


The index s* of the best sample is found by minimizing this distance:s* = argmin_*s*_*d*_*s*_

The final predicted geometry **R**_final_ is then the geometry of the sample with index s*:**R**_final_ = **R**_s_*

Note that we do not access the ground truth TS geometry for selecting the final sample out of the ensemble. This procedure is named AggregateSamples in Algorithm 1. We choose median over mean aggregation, to omit sampling low probability conformers in case of multimodal distributions.

### 2.6 Adaptations

We introduced several adaptations to flow matching and GotenNet for our problem of TS geometry prediction, which we highlight below. The training and inference procedures are described in Algorithm 1.

(1) To incorporate time awareness into the GotenNet architecture for flow matching, we add sinusoidal time embeddings to the initial node and edge features.

(2) To obtain an optimal transport path, we first align the randomly initialized atomic positions with the ground truth positions. We align their center of mass (CoM) and rotationally align the positions using the Kabsch algorithm to find the optimal rotation matrix.^37^ These steps are performed in the Align function of Algorithm 1.

(3) We employ the previously proposed median sample aggregation method during inference.

(4) We initialize node and edge features using CGR-based embeddings. This procedure is described in detail in Algorithm 2.

(5) In an additional experiment we initialize the atomic positions at *t* = 0 with the reactant geometry plus Gaussian noise (*μ* = 0, *σ*^2^ = 0.25). With this modification, we expect to better model chiral TSs.
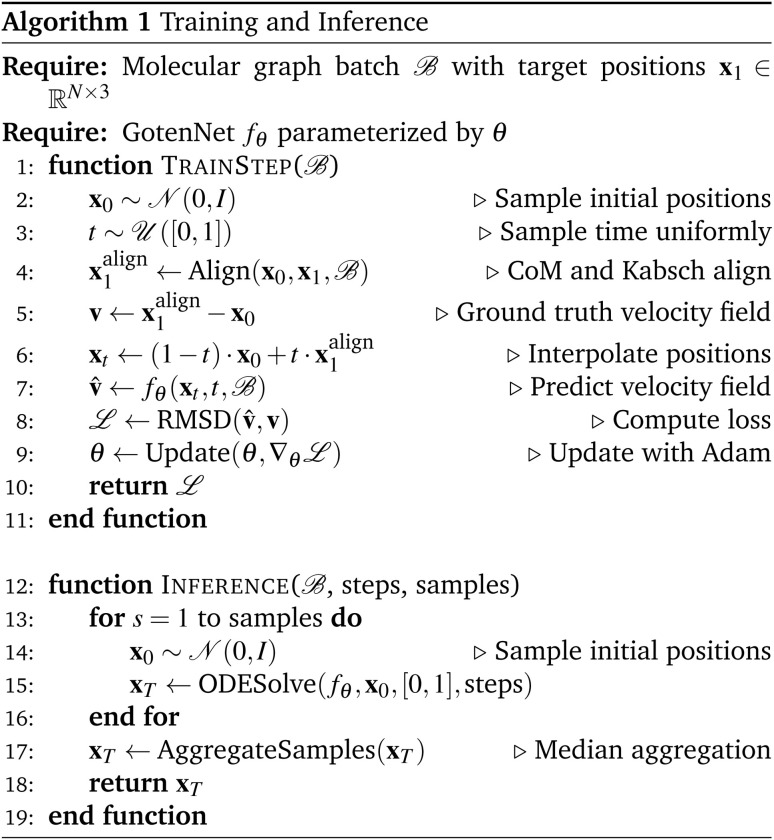

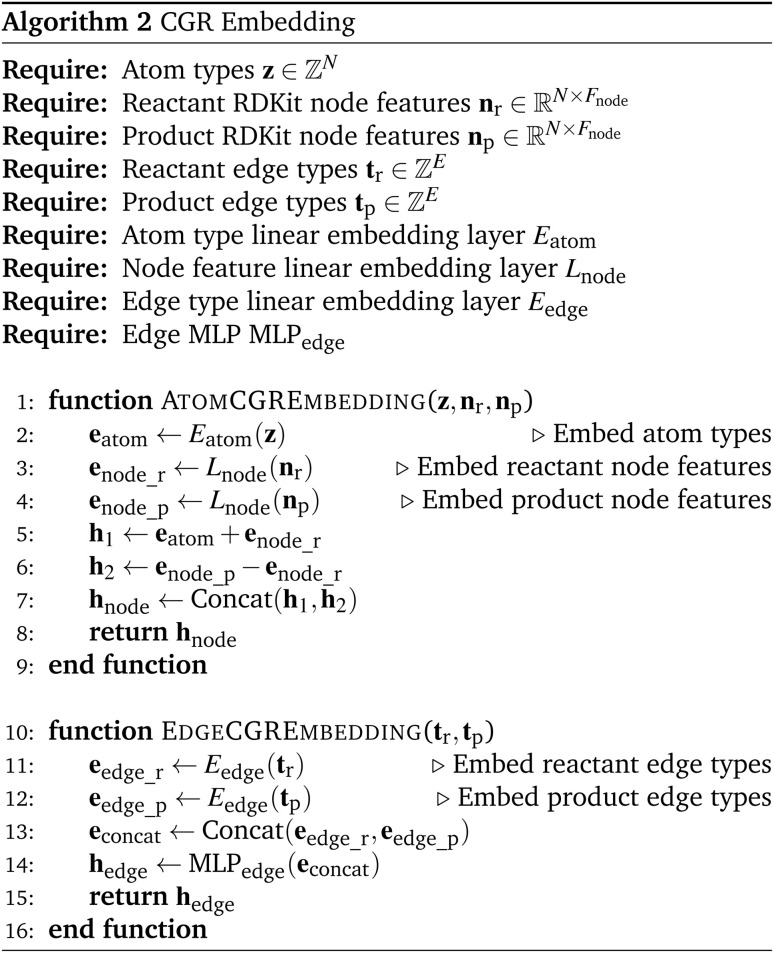


### 2.7 Experimental setup

We evaluate GoFlow on the task of TS geometry prediction and compare it with the current state-of-the-art algorithm TsDiff. For training and testing, we use an NVIDIA A100 GPU.

We perform ablation studies on the number of ODE integration steps, the number of samples to be aggregated during inference, the number of trainable parameters, and three different dataset splitting strategies.

We also evaluate the median absolute deviation in the performance metrics, using an ensemble of 8 models, each trained and evaluated separately using different initialization seeds.

#### 2.7.1 Hyperparameters

GotenNet is trained with the same hyperparameters as reported by the authors.^24^ We add edges between atoms within a cutoff radius of 10 Å, and between atoms that are within a 3-hop neighborhood, following the authors of TsDiff.^18^ For details, see the SI.

#### 2.7.2 Dataset

We evaluate and compare our method to TsDiff on the RDB7 (ref. 9) dataset, which comprises 11 926 gas-phase reactions involving H, C, N, and O with molecules containing up to seven heavy atoms. An evaluation of GoFlow on T1X dataset,^12^ a recomputation of RDB7, is also performed and results reported in detail in the SI. Geometries and vibrational frequencies for RDB7 were obtained at the B97-D3/def2-mSVP and ωB97X-D3/def2-TZVP levels of theory. We split the dataset into training, validation, and test sets in an 80%, 10%, and 10% ratio using different splitting strategies (see Section 2.8). For T1X we use the official split. We use RDKit to extract the following atomic features from SMILES strings: aromaticity, formal charge, hybridization, number of bonds per atom, degree, and ring membership. We adopt the same pre-processing pipeline as Kim *et al.*^18^

#### 2.7.3 Baseline

We compare our method to TsDiff.^18^ TsDiff is a diffusion-based deep learning model designed to predict TS geometries directly from the 2D molecular graphs of the reactant and product, encoded as CGR, *G*_rxn_. They approximate the true TS distribution *p*(*C*_0_|*G*_rxn_) by training a model to reverse a forward diffusion process, where noise is incrementally added to the TS coordinates *C*_*t*_ over discrete time steps *t*. The model learns to predict the score function ∇_*C*t_log *p*(*C*_*t*_|*G*_rxn_). To compare it to our method, we trained it on RDB7.^9^ Contrary to the authors,^18^ we avoid data augmentation in our work.

### 2.8 Evaluation

We split the data into training, validation, and test sets using three splitting strategies and evaluate the model's performance on each. Previous work mostly uses random splits,^15–18^ which is problematic for multiple reasons.^22^

Firstly, it does not account for extrapolation capabilities to out-of-domain samples, which might vary among different model classes, such as when comparing equivariant to invariant models or different generative methods. Secondly, the reactants in the dataset were generated using graph enumeration,^14^ which can result in highly similar reactions ending up in both the training and validation/test sets.

For our ablation studies, we train and test using the random split strategy only, assuming it to be a sufficiently good heuristic for evaluating single-parameter changes on our model. In the following, we summarize the three splitting strategies employed.

(1) Random split. Randomly assign reactions to training, validation, or test set.

(2) Reaction core split. Extract the reaction core (*i.e.*, template), the set of atoms for which adjacent bond types are changed during the reaction, and group all reactions by their common core. Randomly assign a core to the training, validation, or test set. Thus, different sets do not contain reactions of the same core.

(3) Barrier height split. Add reactions in the upper and lower 10% of the barrier heights to the validation or test sets. The rest is added to the training set.

#### 2.8.1 Metrics

The accuracy of the generated geometries is measured using the mean absolute error of the interatomic distances (D-MAE), root mean square deviation (RMSD), and the angle error. For precise definitions, see the SI.

Compared to previous work, we do not report minimum-over-samples (MOS) metrics such as the matching score or the average minimum RMSD (AMR)^18,38–40^ for the evaluation of model performance on RDB7. However we perform an evaluation using a MOS approach in the SI to showcase the future potential of steering-based approaches.^41^ MOS metrics take several independently generated candidate geometries for the same reaction and report the minimum RMSD (or D-MAE) to the ground truth among them. While we evaluate our predictions against the ground truth TS geometry on the test set, we avoid minimum-over-samples metrics in our main evaluation on RDB7 for two reasons:

First, in a real application, the ground truth TS geometry is unknown, so the user cannot identify the “best” sample post hoc. Second, they can give a misleadingly optimistic picture of model accuracy – for example, even a poor model (or random guessing) can achieve an artificially low AMR if enough samples are generated, simply by chance.

Instead, we report metrics computed on the single geometry that would actually be returned to the user in practice, such as the median prediction from multiple samples of the model. This yields error estimates that better reflect the accuracy a chemist could expect when using the model prospectively, without the benefit of knowing which sample is closest to the truth.

Furthermore, we introduce a metric, called steric clash error, with which we aim to identify gross structural deviations in nonbonded interactions. Steric clashes, while barely affecting the D-MAE when most of the molecule's geometry is predicted correctly, result in unrealistically high repulsive energies and thus unrealistic activation energies.^42^ Vost *et al.* demonstrated in recent work^43^ that conditioning diffusion models on conformer quality significantly improves steric clash test results in generated molecules. The recent Boltz-1 architecture for biomolecular structure prediction also uses physical constraint potentials, including steric clash constraints, during inference.^44^ In this work, we use the steric clash error to analyze the results only.

To accomplish this, we use a simplified Lennard-Jones (LJ) interaction potential, where we omitted the London dispersion force term, set *ε* = 0.25 kcal mol^−1^, *σ* = 0.7 Å, and are thus left with 
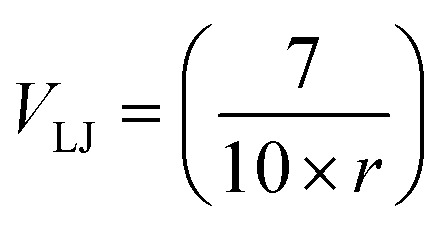
.^12^ We set the steric clash of edges with distances greater than 0.7 Å to zero. Thus, we only consider interatomic distances close to or smaller than the shortest bond lengths (of hydrogen molecules) in our dataset.

## 3 Results and discussion

We first compare GoFlow with TsDiff (Table 1). When using GoFlow with 25 samples and 25 ODE steps, a D-MAE of 0.108 Å, RMSD of 0.18, and an angle error of 3.63° is obtained. This is notably lower than the values for TsDiff (D-MAE of 0.164 Å, RMSD of 0.29, and angle error of 4.77°). GoFlow with 25 samples has an inference time of 125 ms, compared to 1544 ms of TsDiff for a single sample. We also report metrics for GoFlow using 1 sample and 25 ODE steps (see Table 1 first entry) for comparison. In this scenario, GoFlow still outperforms TsDiff for all metrics while being even faster with a run time of 10 ms, more than a hundred times faster than TsDiff. Initializing the atomic positions at *t* = 0 with reactant geometries plus Gaussian noise also improved the RMSD.

**Table 1:** Comparing GoFlow to TsDiff. Performance metrics of GoFlow, with 25 ODE steps and 1 sample (GoFlow-1), 25 ODE steps and 25 samples (GoFlow-25) during inference, and initializing atomic positions with the reactant positions plus noise (GoFlow-25-R, everything else being the same as GoFlow-25). We report the median of 8 training and subsequent test runs with median absolute deviation for GoFlow-25. Metrics are the mean absolute error of interatomic distances (D-MAE), root mean square deviation (RMSD), angle error, and inference runtime per reaction

Method	D-MAE (Å)	RMSD (Å)	Angle (°)	Runtime (ms)
GoFlow-1	0.118	0.20	3.65	10
GoFlow-25	0.108 ± 0.006	0.18 ± 0.005	3.63 ± 0.28	125 ± 0
GoFlow-25-R	0.104 ± 0.002	0.17 ± 0.002	3.56 ± 0.08	130 ± 0
TsDiff	0.164	0.29	4.77	1544

**Fig. 1:**
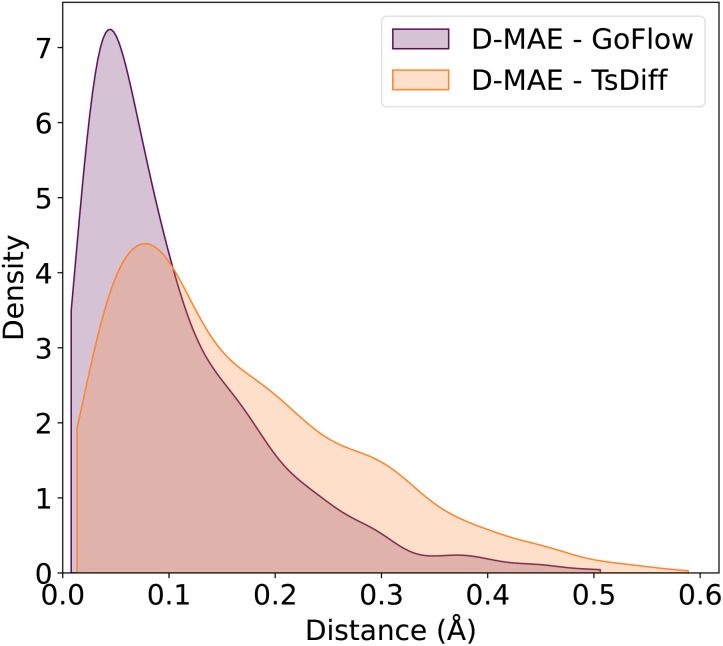
Distributions of the mean absolute error of interatomic distances (D-MAE) in angstroms for GoFlow and TsDiff. Inference performed with 25 ODE solver steps and 25 samples per run.

We also plot the distribution of the obtained D-MAEs on the random split test set in Fig. 1. The bell-shaped distribution features a tail to the right, indicative of outliers with high error that increase the overall D-MAE, and a low peak close to 0.05 Å. In comparison, the low peak of TsDiff is shifted to the right, and the tail is much more pronounced, producing more predictions with a high D-MAE. Fig. 1 thus highlights the improved prediction accuracy reported in the current study, with both a lower number of high D-MAE (low-quality) predictions, and a lower D-MAE for high-quality predictions.

Evaluations of GoFlow on T1X are reported in the SI.

### 3.1 Ablation studies

We found that the number of ODE steps and the number of samples significantly impacted model performance.

The results in Table 2 show that increasing the number of ODE steps and keeping the number of samples at 25, reduces the D-MAE, the angle error, and steric clashes. At 25 ODE steps, the model achieves the lowest D-MAE (0.107 Å), the smallest angle error (3.68°), and a low steric clash error (14 kcal mol^−1^), indicating an optimal balance. Increasing the number of samples and keeping the ODE steps at 25 (Table 3) shows the lowest angle error when using 10 samples and the lowest D-MAE when using 50 samples, while the steric clash remains equally low in both cases. This shows that the ODE steps are crucial to reducing steric clashes.

**Table 2:** Metrics of a model sampling 25 times during inference, aggregated by choosing the sample closest to the median atomic positions of all samples. We report the mean absolute error of the interatomic distances (D-MAE), angle error, and the steric clash score

# ODE steps	D-MAE (Å)	Angle (°)	Steric clash (kcal mol^−1^)
1	1.263	52.56	9351
3	0.182	9.37	2815
5	0.125	5.02	362
10	0.111	3.94	59
25	0.107	3.68	14
50	0.107	3.70	7

**Table 3:** Metrics of the model using 25 ODE steps while varying the number of samples during inference. We report the mean absolute error of the interatomic distances (D-MAE), angle error, and the steric clash score

# Samples	D-MAE (Å)	Angle (°)	Steric clash (kcal mol^−1^)
1	0.119	3.77	4
3	0.117	3.76	12
5	0.113	3.66	22
10	0.107	3.64	21
25	0.107	3.68	14
50	0.105	3.70	14

Furthermore, we conducted ablation studies on the number of trainable parameters of the model by increasing the dimensionality of the atom basis latent space. The results are shown in Table 4. We found the model with 5.2 M parameters to yield the best results. However, the model with 1.4 M parameters still achieved a D-MAE of 0.124, compared to the 0.164 D-MAE of TsDiff, which has 2.7 M trainable parameters.

**Table 4:** Metrics of models during inference, with 25 ODE integration steps and 50 samples, as the dimensionality of the latent space (atom basis) and thus the number of parameters is increased

# Parameters	Atom basis	D-MAE (Å)	Angle (°)
0.4 M	64	0.163	5.94
1.4 M	128	0.124	4.26
5.2 M	256	0.102	3.49
9.3 M	344	0.105	3.56
20.4 M	512	0.114	3.90

### 3.2 Out-of-distribution generalization

**Fig. 2:**
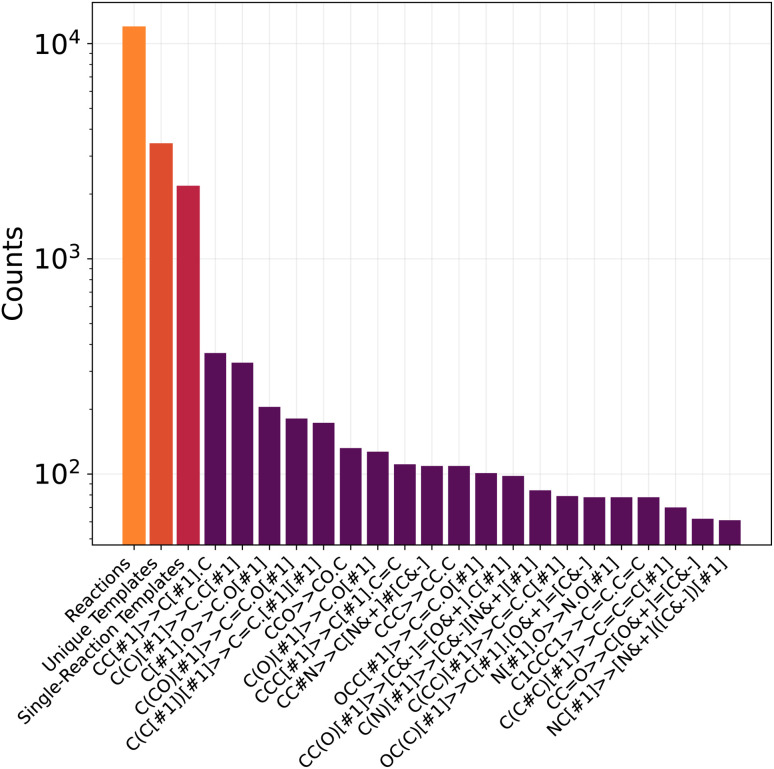
Distributions of the reaction template (core) cluster sizes. The first 3 bars from left to right show the total number of reactions, unique templates, and templates that contain one reaction only.

We trained and tested GoFlow and TsDiff on different dataset splits to evaluate their out-of-distribution generalization capability. The performance of all tested models degraded on the more challenging reaction core split and barrier height split, compared to the random split. The barrier height split was the most challenging for both models. The results are shown in Table 5, and the distribution of reaction cores (templates) is shown in Fig. 2. Importantly, even on the challenging reaction core or barrier height splits, GoFlow outperforms TsDiff, with the D-MAE observed for the more difficult splits with GoFlow still being significantly lower than the performance of TsDiff on random splits. This is especially noteworthy, since the reaction core split requires the model to generalize to unseen reaction types, making it applicable to new reaction types. The results furthermore suggest that it is essential for the trained model to cover the full range of barrier heights expected in a practical setting.

**Table 5:** Performance comparison between GoFlow (with 25 ODE steps, 25 samples) and TsDiff models across three different dataset splitting strategies. In the reaction core split, reactions are clustered by their reaction core, with distinct clusters assigned to either training or validation/test sets. The barrier height split reserves reactions in the top and bottom 10% of barrier heights exclusively for validation/test sets. The random split randomly assigns reactions to either set

Method	Split	D-MAE (Å)	RMSD (Å)	Angle (°)
GoFlow	Random	0.108	0.18	3.63
	Reaction core	0.138	0.22	5.00
	Barrier height	0.149	0.22	5.63
TsDiff	Random	0.164	0.29	4.77
	Reaction core	0.174	0.30	5.51
	Barrier height	0.191	0.32	6.39

### 3.3 Computational efficiency

We performed sampling on a single NVIDIA A100 GPU. For GoFlow, the inference time per reaction on the test set was 0.01 s for 1 sample and 0.13 s for 25 samples, while for TsDiff it was 1.54 s per sample using a batch size of 200 in all cases. This order-of-magnitude speedup is due to using the optimal transport (linear) velocity field for flow matching, which requires substantially fewer sampling steps per reaction and the GotenNet architecture not using higher-order tensor product operations. In our case, it is 625 forward passes per reaction for the best performing model with 25 ODE solver and 25 sampling steps, compared to the 5000 steps of TsDiff. We also note that we did not parallelize the sampling steps of GoFlow, which would further speed up inference time significantly.

### 3.4 Error modalities

**Fig. 3:**
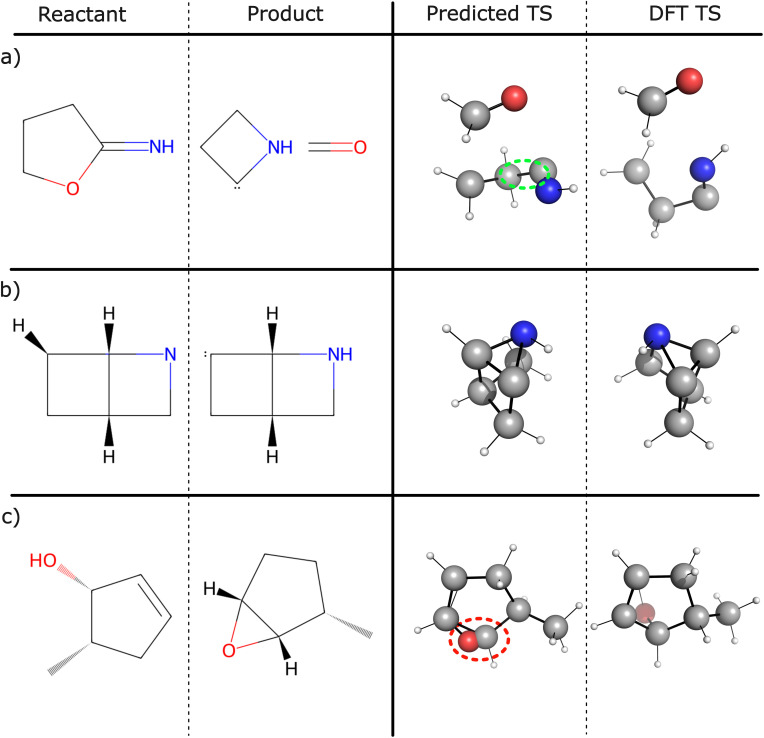
Visualization of three types of error modalities. (a) High D-MAE: predicted structure with a D-MAE of 0.5 Å, resulting from an inaccurate dihedral angle (circled in green) and inaccurate distances between formaldehyde and azacyclobutyl-2-ylidene. Despite these inaccuracies, the steric clash remains low at 1.4 kcal mol^−1^. (b) Chirality: the model incorrectly predicts the opposite enantiomer. This error type does not impact D-MAE values, as distances are preserved under reflections. (c) Steric clash: predicted structure with a severe steric clash score of almost 7000 kcal mol^−1^, despite a low D-MAE of 0.16 Å. While most of the geometry is accurately predicted, the oxygen atom is positioned too close to a carbon atom, which renders the structure physically implausible.

We highlight three error modalities in Fig. 3.

#### 3.4.1 High D-MAE

In Fig. 3(a) we show an example with a high D-MAE of 0.5 Å. We observe an inaccurate dihedral angle when comparing the GoFlow predicted TS with the DFT TS. The distances between the two molecules in the TS are also inaccurately predicted. Although the D-MAE is high, a very low steric clash value of 1.4 kcal mol^−1^ is obtained.

#### 3.4.2 Chirality

Fig. 3(b) shows a chiral reaction for which the wrong enantiomer TS was predicted, resulting in a high RMSD.

#### 3.4.3 Steric clash

In Table 2, we see that increasing the number of ODE steps drastically reduces the steric clash error. Fig. 3(c) shows an example molecule with a severe steric clash of almost 7000 kcal mol^−1^, but a low D-MAE of 0.16 Å. Although most of the geometry is accurately predicted, an oxygen atom is positioned too close to a carbon atom, which results in low D-MAE but high steric clash. This renders the structural information unsuitable for downstream applications, such as barrier height predictions,^29^ which would yield unrealistically high energy values.

In particular, these errors show that relying solely on metrics such as the D-MAE for performance evaluation, which is currently common practice,^15,18^ is insufficient. D-MAE is insensitive to steric clashes, and because distances are preserved under reflections, it also fails to capture chirality errors.

Moreover, the identified error modalities appear to occur more frequently in reactions that trained chemists would classify as unlikely or unphysical. Examples include reactions involving unstabilized carbenes (Fig. 3(a) and (b)), energetically unfavorable polycycles (Fig. 3(c)), or zwitterions, the latter being present in unusually many products in the dataset. This observation highlights the importance of chemically informed dataset curation for developing robust TS prediction models.

### 3.5 Downstream applications

The predicted geometries can be used as input to downstream applications, for example, as guess structures for quantum mechanical calculations of the TS or for predicting reaction properties.

#### 3.5.1 Transition state optimization

To assess the potential of GoFlow for initializing TS optimizations instead of running expensive reaction path searches such as Nudge-Elastic Band (NEB) searches, we performed quantum mechanical optimization of the predicted TS geometries for the first 300 reactions in our test set. All calculations were run using ORCA 6.0 at the ωB97X-D4/def2-TZVP level of theory. TS optimization resulted in 280 successfully optimized geometries with exactly one imaginary vibrational frequency for GoFlow, compared to 269 for TsDiff.

The optimized geometries of GoFlow were significantly lower in energy, with the mean of individual differences in the TS energy being 4.76 kcal mol^−1^, and requiring less optimization steps to reach the TS (median of 26 for GoFlow *versus* 35 for TsDiff). In subsequent intrinsic reaction coordinate (IRC) calculations, 225 of the 280 optimized geometries for GoFlow converged to the correct reactants and products, and 216 of 269 for TsDiff. Matches in those reactants and products were determined by converting the IRC geometries to SMILES strings and comparing those. These results, summarized in Table 6, indicate that GoFlow provides effective starting points for quantum mechanical TS optimization, and outperforms TsDiff.

**Table 6:** Quantum chemical validation for the first 300 reactions in the random split test set. We report the following metrics: success rate, which is the percentage of reactions for which the optimized geometry has a single imaginary frequency. IRC Match, which is the percentage of those single imaginary frequency geometries whose IRC calculations resulted in the correct reactants and products in terms of their SMILES strings. Optimization cycles, which is the median number of geometry optimization cycles

Method	Success rate	IRC match	Optimization cycles
GoFlow	94%	80.4%	25
TsDiff	92%	80.3%	35

Moreover, out of the 225 IRC validated geometries by GoFlow, 136 had lower single-point energies than their reference structure. Of those 136, 21 had an energy that was more than 0.1 kcal mol^−1^ lower than the reference energy, and for 13 it was more than 1 kcal mol^−1^ lower. In the SI we show one such example, as well as the distributions of atomic force magnitudes.

The distribution of atomic force magnitudes of the non-optimized TS structures is highly similar for both methods. GoFlow has slightly more atoms at the low-force end of the distribution. This is consistent with GoFlow reaching converged TSs slightly more often and with fewer optimization cycles.

#### 3.5.2 Barrier height prediction

Karwounopoulos *et al.*^45^ recently showed significant improvements in barrier height prediction on the RDB7 and RGD1 (ref. 8) datasets, when using auxiliary 3D information of the TS as input to their model, in addition to the 2D CGR. Those 3D geometries were generated with either TsDiff or GoFlow and encoded using MACE^35^ descriptors. Using GoFlow geometries compared to TsDiff resulted in improved barrier height predictions for both datasets.

## 4 Conclusion

We proposed GoFlow, an E(3)-equivariant flow-matching-based method for predicting transition state geometries, using only the 2D reaction graph as input. It drastically increased the inference speed while significantly improving the quality of the generated geometries compared to existing methods. We analyzed out-of-distribution performance and showed that, similarly to reaction property prediction methods, performance decreases for challenging dataset splits. While GoFlow provides better out-of-distribution performance than previous approaches, our results still indicate a large potential for future work to improve upon. Nevertheless, we successfully demonstrated its potential to create guess structures for quantum-mechanical transition state optimizations, bypassing full reaction path searches, and to serve as input to machine learning models for reaction property prediction.

## Author contributions

LG: conceptualization, formal analysis, investigation, methodology, project administration, software, validation, visualization, writing – original draft. KM: formal analysis, investigation, software, validation, writing – review & editing. JK: validation, visualization, writing – review & editing. MK: data curation, investigation, formal analysis, validation, writing – review & editing. EH: conceptualization, funding acquisition, project administration, supervision, writing – review & editing.

## Conflicts of interest

There are no conflicts to declare.

## Supplementary Material

DD-004-D5DD00283D-s001

## Data Availability

The reaction dataset used in this study is freely available at https://doi.org/10.1038/s41597-022-01529-6. The GoFlow code is freely available at https://github.com/heid-lab/goflow. All data as well as the archived version of GoFlow to reproduce results is available on Zenodo 10.5281/zenodo.17251208. Supplementary information: results for Transition1X, details on the metrics, quantum mechanical analysis, details on hyperparameters. See DOI: https://doi.org/10.1039/d5dd00283d.
