# Supplementary material for: GoFlow: efficient transition state geometry prediction with flow matching and E(3)-equivariant neural networks
Source: Digit Discov. 2025 Oct 21;4(12):3492–501. doi: 10.1039/d5dd00283d (PMC12580847; doi:10.1039/d5dd00283d)
Supplement: DD-004-D5DD00283D-s001 [file DD-004-D5DD00283D-s001.pdf]

Supplementary Information (SI) for Digital Discovery.
This journal is © The Royal Society of Chemistry 2025

Supplementary Information:
GoFlow: Efficient Transition State Geometry Prediction with Flow Matching and E(3)-Equivariant Neural Networks

Leonard Galustian, Konstantin Mark, Johannes Karwounopoulos, Maximilian Kovar, and Esther Heid[a)]

Institute of Materials Chemistry, TU Wien, A-1060 Vienna, Austria

[a)]Electronic mail: esther.heid@tuwien.ac.at

A. Transition1X Results

For comparison with concurrent and future work, we evaluated the performance of GoFlow on the T1X dataset[2] and report the results in Table S1. We also report results of React-OT[1] in that table. We note that their method is not directly comparable to ours or TsDiff, since it does not predict distributions of transition states but is deterministic.

When choosing the best generated sample out of all samples (GoFlow-{10,25}-B in Table S1), GoFlow is able to outperform React-OT on T1X while also being stochastic. Note that this represents an idealized scenario, since in practice we do not observe the ground truth structure and thus cannot select the best out of all samples. However, we observe that generative models could predict significantly more accurate structures, given a-priori information such as an energy function to help select the most relevant structure from all generated ones. One promising method to achieve this is based on inference-time steering towards an energy-tilted posterior.[3]

Method	D-MAE (Å)	RMSE (Å)	Bond Angle (°)	Runtime (ms)
GoFlow-10	0.109	0.18	4.41	40
GoFlow-10-R	0.100	0.17	4.12	44
GoFlow-10-B	0.070	0.10	2.78	40
GoFlow-25-B	0.062	0.08	2.46	90
React-OT	-	0.10	-	50-400

Table S1. Evaluating GoFlow on the Transition1X dataset, with 25 ODE steps and 10 samples with a Gaussian initial distribution (GoFlow-10), and initializing atomic positions with the reactant positions plus noise (GoFlow-10-R, everything else being the same as GoFlow-10). We also show results where we assumed a-priori knowledge of which sample is closest to the reference structure: GoFlow-10-B and GoFlow-25-B with 10 and 25 samples respectively, and an initial Gaussian distribution. We also report the performance of React-OT[1]. Metrics are the mean absolute error of interatomic distances (D-MAE), root mean square error (RMSE), bond angle error, and inference runtime per reaction. We use the official dataset split provided by the authors.[2], which differs from the split used by React-OT.

B. Metrics

Below we define the D-MAE, RMSD, and bond angle error metrics used to evaluate the methods.

The D-MAE is defined as

$$\text{D-MAE}(\mathbf{R}^{\text{pred}}, \mathbf{R}^{\text{gt}}) = \frac{2}{N(N-1)} \sum_{i<j} \left| d_{ij}^{\text{pred}} - d_{ij}^{\text{gt}} \right|, \tag{1}$$

where N is the number of atoms, and d_{ij} the Euclidean distance between atoms i and j.

The RMSD is defined as

$$\text{RMSD}(\mathbf{R}^{\text{pred}}, \mathbf{R}^{\text{gt}}) = \sqrt{\frac{1}{3N} \sum_{i=1}^{N} \left[(r_{x,i}^{\text{pred}} - r_{x,i}^{\text{gt}})^2 + (r_{y,i}^{\text{pred}} - r_{y,i}^{\text{gt}})^2 + (r_{z,i}^{\text{pred}} - r_{z,i}^{\text{gt}})^2 \right]} \tag{2}$$

where $\mathbf{r}^i \in \mathbb{R}^3$ are the Cartesian coordinates of atom i. Note that, following concurrent work,[1] we ignore chirality for RMSD calculations.

The angle error is defined as

$$\text{AngleError}(\mathbf{R}^{\text{pred}}, \mathbf{R}^{\text{gt}}) = \frac{1}{M} \sum_{(i,j,k) \in \mathcal{A}} \left| \theta_{ijk}^{\text{pred}} - \theta_{ijk}^{\text{gt}} \right| \cdot \frac{180}{\pi}, \tag{3}$$

where

$$\theta_{ijk} = \arccos\left(\frac{(\mathbf{r}_i - \mathbf{r}_j) \cdot (\mathbf{r}_k - \mathbf{r}_j)}{\|\mathbf{r}_i - \mathbf{r}_j\|_2 \, \|\mathbf{r}_k - \mathbf{r}_j\|_2} \right), \tag{4}$$

and M is the number of angles θ_{ijk}, defined by the set of all triplets of atoms $(i, j, k) \in \mathcal{A}$ in the structure.

C. Quantum Mechanical Analysis

The distributions of the force magnitudes of each atom of the 300 non-optimized predicted TS structures of GoFlow and TsDiff are shown in Figure S1. Subsequent quantum mechanical calculations were performed on these TSs. This included saddle-point optimizations and subsequent IRC path calculations to validate if the saddle-point connected the expected reactants and products of the reaction. For those validated reactions, we observed several

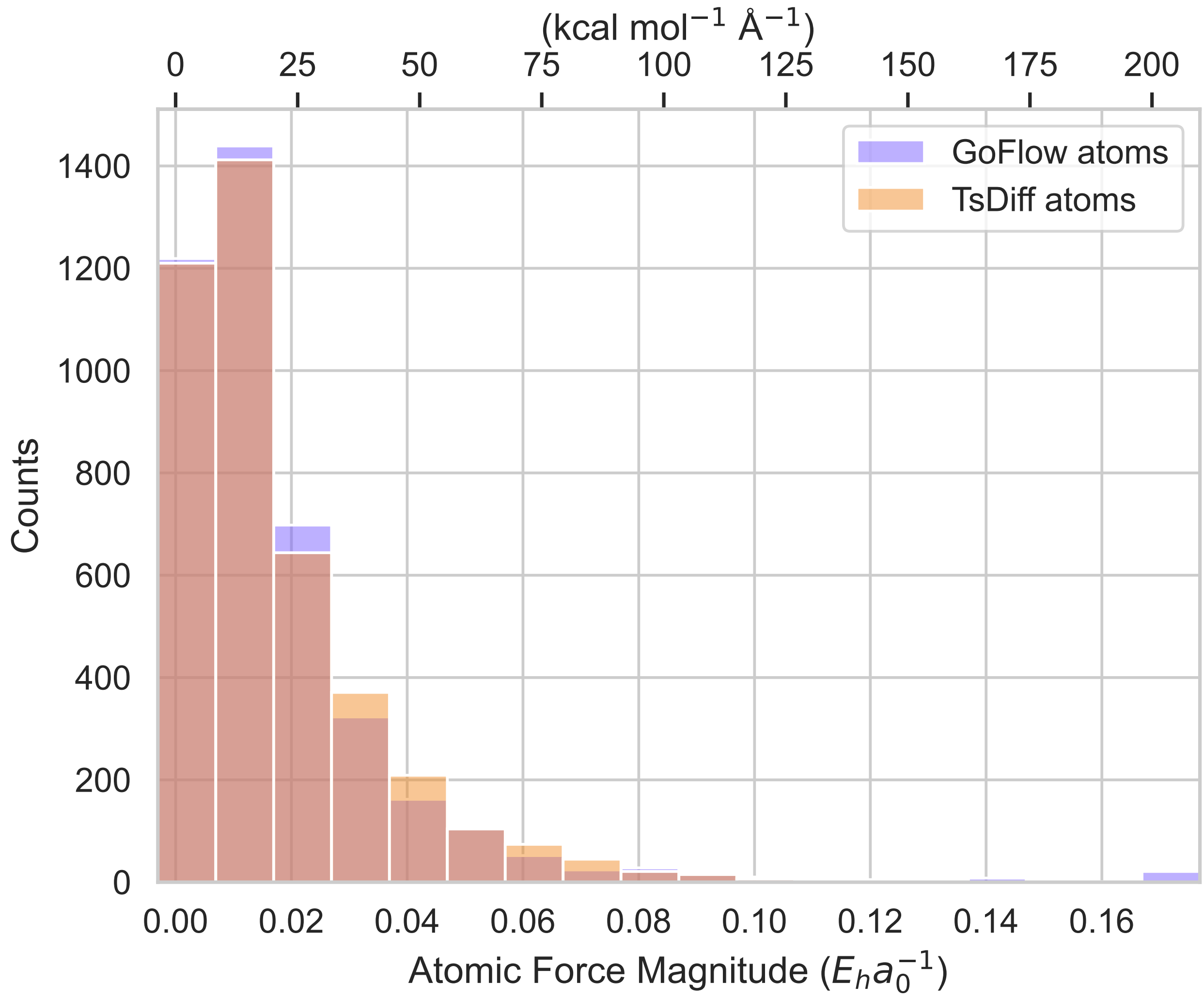

Figure S1. Distribution of the force magnitudes of each atom of all 300 non-optimized predicted TS structures of GoFlow and TsDiff from RDB7 (for which subsequent quantum mechanical calculations were performed).

with lower TS energies compared to the reference TS. An example of this is shown in Figure S2.

D. Hyperparameters

Table S2 summarizes the main hyperparameters used in our experiments. We used the hyperparameters provided by TsDiff and GotenNet for data preprocessing, training, and evaluation. We did not perform optimizations on those, except for our ablation study on model size, where we evaluated the effect of varying the hidden atom features (atom basis)

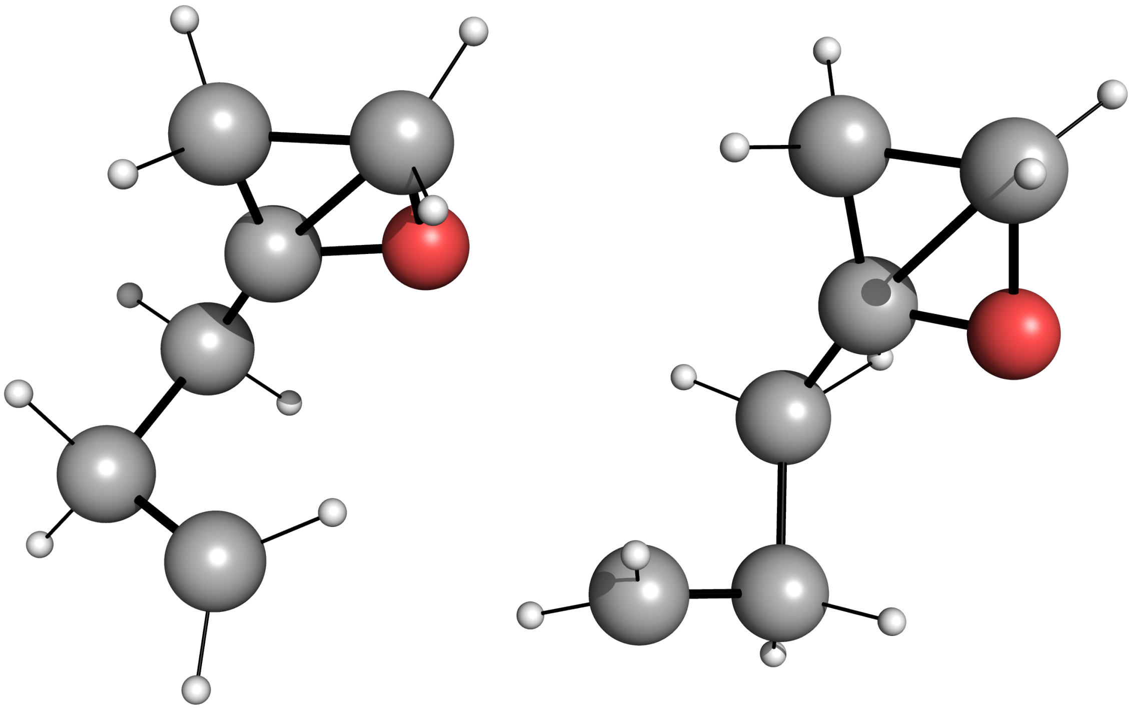

Figure S2. Predicted and subsequently optimized TS structure on the left hand side (RDB7 dataset). Reference structure on the right. The predicted structure has a 15 kcal/mol lower energy. The greatest energy improvement out of all IRC validated reactions which were analyzed for this figure.

size. We refer the reader to those papers[4,5] or our source code for more details.

**Hyperparameter**	**Value**
Learning rate	5×10^{-4}
Learning rate decay	0.8 (patience = 5)
Weight decay	0.01
EMA decay	0.9
Batch size	200
Max. epochs	650
Early stopping patience	25
Gradient clipping	5.0
Number of flow ODE steps	25
Hidden atom features	256
Hidden output units	64
Number of interaction layers	3

Table S2. Main hyperparameters used in training and testing GoFlow for both RDB7 and Transition1X. We employed the default hyperparameter values from TsDiff and GotenNet, and only performed ablation studies on the number of flow ODE steps and number of aggregation samples.

REFERENCES

[1]C. Duan, G.-H. Liu, Y. Du, T. Chen, Q. Zhao, H. Jia, C. P. Gomes, E. A. Theodorou, and H. J. Kulik, Nature Machine Intelligence **7**, 615 (2025).

[2]M. Schreiner, A. Bhowmik, T. Vegge, J. Busk, and O. Winther, Scientific Data **9**, 779 (2022), publisher: Nature Publishing Group.

[3]K. Mark, L. Galustian, M. P.-P. Kovar, and E. Heid, "Feynman-Kac-Flow: Inference Steering of Conditional Flow Matching to an Energy-Tilted Posterior," (2025), arXiv:2509.01543 [cs].

[4]S. Aykent and T. Xia, in *The Thirteenth International Conference on Learning Representations* (2025).

[5]S. Kim, J. Woo, and W. Y. Kim, Nature Communications **15**, 341 (2024).
